# The Effect of Dietary Supplementation with Probiotic and Postbiotic Yeast Products on Ewes Milk Performance and Immune Oxidative Status

**DOI:** 10.3390/jof9121139

**Published:** 2023-11-25

**Authors:** Christos Christodoulou, Alexis Skourtis, Panagiota Kyriakaki, Fotis Fokion Satolias, Dimitris Karabinas, Maxime Briche, Nizar Salah, George Zervas, Alexandros Mavrommatis, Eleni Tsiplakou

**Affiliations:** 1 Laboratory of Nutritional Physiology and Feeding, Department of Animal Science, School of Animal Biosciences, Agricultural University of Athens, Iera Odos 75, GR-11855 Athens, Greece; c.christodoulou@aua.gr (C.C.); alexis_skourtis@hotmail.com (A.S.); kyriakaki@aua.gr (P.K.); satoliasf@gmail.com (F.F.S.); vdkarabinas@vkarabinas-sa.gr (D.K.); gzervas@aua.gr (G.Z.); mavrommatis@aua.gr (A.M.); 2 Phileo by Lesaffre, 59700 Marcq en Baroeul, Nord, France; m.briche@phileo.lesaffre.com (M.B.); n.salah@phileo.lesaffre.com (N.S.)

**Keywords:** dairy ewes, peripartum period, live yeast, mannan-oligosaccharides, organic selenium, milk quality, B-HBA, oxidative status, innate immunity, probiotic, postbiotic

## Abstract

The administration of yeast products as feed additives has been proven to beneficially affect animal productivity through energy, oxidative, and immune status improvement. This study evaluated a combination of *Saccharomyces cerevisiae* live yeast (LY) with yeast postbiotics (rich in mannan-oligosaccharides (MOS) and beta-glucans) and selenium (Se)-enriched yeast on ewes’ milk performance and milk quality, energy and oxidative status, and gene expression related to their immune system during the peripartum period. Ewes were fed a basal diet (BD; F:C = 58:42 prepartum and 41:59 postpartum) including inorganic Se (CON; *n* = 27), the BD supplemented with a LY product, and inorganic Se (AC; *n* = 29), as well as the combination of the LY, a product of yeast fraction rich in MOS and beta-glucans, and organic-Se-enriched yeast (ACMAN; *n* = 26) from 6 weeks prepartum to 6 weeks postpartum. The β-hydroxybutyric acid concentration in the blood of AC and ACMAN ewes was lower (compared to the CON) in both pre- and postpartum periods (*p* < 0.010). Postpartum, milk yield was increased in the AC and ACMAN Lacaune ewes (*p* = 0.001). In addition, the activity of superoxide dismutase (*p* = 0.037) and total antioxidant capacity (*p* = 0.034) measured via the 2,2-Azino-bis (3-ethylbenzthiazoline-6-sulfonic acid) (ABTS) method was increased in the blood plasma of the ACMAN postpartum. Higher ABTS values were also found (*p* = 0.021), while protein carbonyls were reduced (*p* = 0.023) in the milk of the treated groups. The relative transcript levels of *CCL5* and *IL6* were downregulated in the monocytes (*p* = 0.007 and *p* = 0.026 respectively), and those of *NFKB* were downregulated in the neutrophils of the ACMAN-fed ewes postpartum (*p* = 0.020). The dietary supplementation of ewes with yeast postbiotics rich in MOS and beta-glucans, and organic Se, improved energy status, milk yield and some milk constituents, and oxidative status, with simultaneous suppression of mRNA levels of proinflammatory genes during the peripartum period.

## 1. Introduction

The peripartum period of ruminants is a challenging phase since nutrient requirements rise to support both fetal growth and the production of colostrum and milk [1]. During this period, ruminants face a negative energy balance (NEB) since both appetite and rumen volume are suppressed while the energy and nutrient demands are exponentially increasing. The impairment of organisms’ homeostasis due to the NEB may lead to dysregulation in the immune system and oxidative status [2,3,4,5]. Hence, metabolic, and infectious diseases such as enterotoxemia, ketosis, and mastitis may arise [3,6]. In addition, reactive oxygen species (ROS) are produced at higher rates due to the rapid growth of the fetuses and the high sheep prolificacy [7]. After gestation and throughout early lactation, there are high energy and nutrient requirements. To support this, there is increased consumption of concentrates with rapidly fermentable carbohydrates, which alters the rumen microbiome (dysbiosis) [5], increasing the risk of metabolic disorders such as ruminal acidosis [8,9].

To tackle these metabolic issues, the administration of feed additives such as probiotics became popular and are used in ruminant rations, especially since their official approval as feed additives in EU [10]. These additives prevent disturbances of the rumen microbial environment and support rumen anaerobiosis [11,12]. Improving feed conversion as well as energy and nutrient availability in ruminants during the peripartum period not only improves ruminants’ performance (milk yield and chemical composition) but also reduces the immune–stimulatory response by limiting lipo-mobilization metabolites [13]. Although there is ample evidence to support the mode of action of live yeasts (LY) in ruminants, only scarce information exists regarding their combination with postbiotics (yeast’s cell walls and structural components) [14,15,16]. It has been well stated that mannan oligosaccharides (MOS) and beta-glucans, which can be found in products from yeast fractions, can prevent the attachment of harmful bacteria in the gastrointestinal tract, thus benefiting the intestinal mucosa and providing immune defense [17], while beta-glucans also pose immunomodulatory properties by enhancing the animals’ innate immunity [18]. In this context, supplementing cattle’s concentrate diets with either yeast or MOS resulted in a ruminal pH increase, while LPS translocation into the bloodstream and blood serum amyloid A concentration were decreased [19]. Moreover, selenium (Se), as a selenocysteine, is another feed additive with a well-established role during the peripartum period, neutralizing ROS created by both high metabolic rates and metabolite damage due to NEB through the action of glutathione peroxidase (GSH-Px) [20]. However, different Se sources may demonstrate variable bioavailability rates in ruminants and may trigger antioxidant and immune responses to a different extent [21,22].

In addition to the critical transition period and its impact on the physiology of small ruminants, the intensification of production systems could hide many other challenges. Small ruminants are generally fed in groups, with a large number of individuals constituting a group. At the same time, high physical and performance variability is observed (compared to cattle herds) due to the simultaneous breeding of several breeds in the same herd. Additionally, the ration is generally provided in two equal meals in a constant quantity based on the group’s average nutritional needs. Considering the characteristics mentioned above, individuals may be over or undernourished, worsening the condition generated by the transition period, and adding another nutritional challenge. Thus, this study aims to investigate the combination of LY, yeast cell wall fraction, and highly bioavailable Se on ewes’ performance and physiology during the peripartum period under commercial conditions in a feeding schedule with a constant amount of feed in a diversified flock, typical of the sheep farms in Greece.

## 2. Materials and Methods

### 2.1. Animal Ethics

The study was conducted from November 2021 to March 2022 on a commercial intensive dairy sheep farm in the region of Chiliomodi Korinthias, Greece. Animal handling procedures were with respect to the guidelines of the European Union Directive on the defense of animals used for scientific purposes following directive EU 63/2010 and the Council of the European Union 2010.

### 2.2. Animals and Husbandry

A total of 120 multiparous 3- to 4-year-old dairy Chios- and Lacaune-breed ewes (*Ovis aries*) (out of a herd with more than 300 homogenous ewes), were selected around mid-November 2021 (expected to lamb in late December 2021 to early January 2022) based on their previous milk performance (2020–2021) and their parity number. At approximately 6 weeks before parturition, ewes were divided into three homogeneous dietary groups of 40 ewes each based on their body weight (BW; 73.95 ± 3.37 kg), parity number (2–3), and previous lactation’s fat-corrected milk yield.

### 2.3. Dietary Treatments

The ewes were fed a basal diet (BD) consisting of concentrate, alfalfa hay, and oat hay (Table 1). Before parturition, to the basal diet (BD; F:C = 58:42) of the control group (CON), 0.30 mg of inorganic Se/kg concentrate was added. To the BD of the second group (AC) 1 g ActiSaf^®^ (CNCM I-4407, 10^10^ CFU/g, ActiSaf^®^; Phileo by Lesaffre, Marcq-en-Barœul, France) and 0.30 mg inorganic Se/kg concentrate were added. To the BD of the third group (ACMAN) 1 g ActiSaf^®^/kg concentrate (CNCM I-4407, 1010 CFU/g, ActiSaf^®^; Phileo by Lesaffre, Marcq-en-Barœul, France), 2 g Safmannan^®^/kg concentrate (concentration of ≥20% of mannans and ≥20% of beta-glucans; Phileo by Lesaffre, Marcq-en-Barœul, France), and 0.20 mg organic Se/kg concentrate (SelSaf^®^; Phileo by Lesaffre, Marcq-en-Barœul, France) were added (Table 1).

After parturition, the CON was fed with a BD with F:C = 41:59, in the concentrate of which 0.30 mg of inorganic Se/kg was contained. To the concentrate of BD of the second group (AC), 0.5 g ActiSaf^®^ (CNCM I-4407, 10^10^ CFU/g, ActiSaf^®^; Phileo by Lesaffre, Marcq-en-Barœul, France) and 0.30 mg inorganic Se/kg were added. To the concentrate of BD of the third group (ACMAN), 0.5 g ActiSaf^®^/kg concentrate (CNCM I-4407, 10^10^ CFU/g, ActiSaf^®^; Phileo by Lesaffre, Marcq-en-Barœul, France), 1 g Safmannan^®^/kg concentrate (concentration of ≥20% of mannans and ≥20% of beta-glucans; Phileo by Lesaffre, France), and 0.20 mg organic Se (SelSaf^®^; Phileo by Lesaffre, Marcq-en-Barœul, France)/kg were added (Table 1).

Each dietary group was further divided into two sub-groups (6 sub-groups in total) based on the animal’s breed, aiming to assess animal performance traits separately even if nutritional requirements were calculated at the whole-flock level, and diets were provided in the same quantity for both breeds with the aim of simulating a common commercial challenge, as explained above. Rations were formulated based on NRC 2007 to meet ewes’ requirements in the prepartum period and early lactation and were isonitrogenous and isocaloric. Due to some unexpected issues during late pregnancy or early lactation (e.g., mastitis, abortions, deaths), few ewes were withdrawn from the trial; thus, the CON group consisted of 27 ewes in total (12 Chios and 15 Lacaune), while AC and ACMAN groups consisted of 29 (16 Chios and 13 Lacaune) and 26 (14 Chios and 12 Lacaune), respectively. The experiment ended 6 weeks after lambing.

### 2.4. Feed Sample Analysis

Samples of the alfalfa hay, oat hay, and concentrate were analyzed for crude protein (Official Method 7.016) according to the Association of Official Analytical Chemists (AOAC) [23] using a Kjeldahl Distillation System (FOSS Kjeltec8400, Hillerød, Demark) while ether extract was determined according to Soxhlet [24]. The dry matter content was measured by drying the sample at 103 °C overnight. The ash-free neutral detergent fiber treated with amylase (aNDFom) and acid detergent fiber (ADF) were analyzed according to Van Soest [25] (Table 2).

### 2.5. Milk Sample Collection

Ewes were milked three times per day (at 7 am, 1 pm, and 8 pm) with a milking machine equipped with a digital milk meter and an electronic identification system (Sylco, Sindos, Greece). Milk yield was recorded daily, and software was set to provide the weekly averages. Milk samples were collected from each ewe weekly (at 7, 14, 21, 28, 35, and 42 days from parturition; days in milk) for 6 weeks with sampling bottles (Sylco, Sindos, Greece) of 200 mL suitably for the milking parlor to receive a representative sample of the milked quantity.

### 2.6. Milk Chemical Composition

Fat, protein, lactose, total solids, and total solids no-fat were determined in individual milk samples through IR spectrometry (MilkoScan 120; FOSS, Hillerød, Demark). Casein content was analyzed according to the reference method, ISO 17997-1/IDF [26] using a FOSS Kjeltec™ 8400 Analyzer Unit and a FOSS Digestion System DT220 (FOSS, Hillerød, Denmark).

### 2.7. Blood β-Hydroxybutyric Acid (B-HBA)

On average, 15–20 days before the expected parturition, blood β-hydroxybutyric acid (B-HBA) was measured individually for each ewe before the morning feeding. Two to three weeks after lambing, B-HBA was again measured individually for each ewe. An electrochemical capillary blood-monitoring system (FreeStyle Precision Neo, Abbott Laboratories Hellas S.A.) and the matching individual foil-wrapped test strips for B-HBA were used to measure blood ketone concentrations. In Chios ewes, this method of B-HBA assessment had a 98.4% accuracy rate for predicting both pregnancies from toxemia and ketosis [27]. The B-HBA concentration was recorded after a drop of blood was applied to the designated area after the test strip had been inserted into the instrument.

### 2.8. Antioxidant Status and Immune Transcriptional Profile

Aiming to validate our previous results on the impact of LY (AC) on Chios ewes peripartum period [13] and further explore its combination with yeasts’ mannans, beta-glucans, and organic Se (ACMAN), we focused again on Chios ewes only. Additionally, given that the commercial farm was far away from the University facilities, cells should be isolated immediately (without any storage) from whole blood using a long-term assay, and a maximum of only 30 samples could be handled as a batch, this sampling procedure was limited to eight samples per dietary group (24 samples/sampling time) obtained by only the Chios ewes. More specifically, 8 Chios ewes of each group with comparable milk performance (fat-corrected milk yield 6% (FCM6%) (CON: 2.87 kg SD: ±0.8; AC: 2.90 kg SD: ±0.7; ACMAN: 2.90 kg SD: ±0.4), ages (parity), and same lactation stage (up to 3 days deviation between animals) were selected for determining the oxidative status of both blood and milk and for immune system gene transcriptional profile. Milk samples were collected (as mentioned above 2.5.) in the 3rd and 6th weeks postpartum and stored at −80 °C. Blood samples were also collected before the morning feeding (0700 h) at the same time points in heparin-contained tubes for cell extraction and plasma isolation.

Twenty (20) mL of whole blood was directly transferred to heparin-containing tubes (17 units heparin/mL; BD Vacutainer, Plymouth, UK). Afterward, 10 mL of blood was centrifuged (SL16R, Thermo Fisher Scientific, Waltham, MA, USA) at 4000 rpm for 15 min at 4 °C to separate plasma from the cells. The assays for the determination of antioxidant enzyme activities, the total antioxidant capacity, as well as the oxidative stress biomarkers were carried out using a UV/V spectrophotometer (GENESYS 180, Thermo Fisher Scientific, Waltham, NA, USA) and were comprehensively described by our previous work [28].

For the isolation of monocytes and neutrophils, 10 mL blood samples were used using a Histopaque density gradient (Sigma-Aldrich Co., St. Louis, MI, USA) as described by Tsiplakou et al. [29]. Firstly, blood monocytes and neutrophils were isolated, and then total RNA was extracted. Pure RNA (500 ng) from 96 individual (monocytes (48: 24 ewes × 2 sampling time) and neutrophils (48: 24 ewes × 2 sampling time)) samples was reverse-transcribed with the PrimeScript First Strand cDNA Synthesis Kit (Takara, Shiga, Japan) according to the manufacturer’s instructions using a mix of random hexamers and oligo-dT primers. Primers used were previously designed by Tsiplakou et al. [29] using Geneious Prime 2023.2 (Biomatters, New Zealand) (Additional file: Table S1). The relative expression levels of the target genes were calculated as (1 + E)^−ΔCt^, where ΔCt is the difference between the geometric mean of the two housekeeping genes’ Cts and the Ct of the target gene, and the primer efficiency (E) is the mean of each amplicon’s efficiency per primer, which was calculated by employing the linear regression method on the log (fluorescence) per cycle number (ΔRn) using the LinRegPCR software (version 11.0). Glyceraldehyde 3-phosphate dehydrogenase (GAPDH) and tyrosine 3-monoxygenase/tryptophan 5-monooxygenase activation protein, zeta polypeptide (YWHAZ) were used as housekeeping genes to normalize the cDNA template concentrations [30].

### 2.9. Statistical Analysis

The statistical analysis was carried out using IBM SPSS Statistics for Windows (IBM Corp., Released 2016, IBM SPSS Statistics for Windows, Version 24.0, Armonk, NY, USA).

A general linear model for repeated measures analysis of variance (ANOVA) was followed to assess the effect of the dietary treatment on ewes’ milk performance, milk chemical composition, body weight, and B-HBA in each breed (Chios and Lacaune) separately. Additionally, the same model was applied for immune oxidative variables in Chios ewes. The dietary treatments (D) (CON, AC, and ACMAN) were defined as the fixed factor and the sampling time (S) as the repeated measure. The interaction between dietary treatment and sampling time (D × S) was also evaluated in accordance with the following model:

Yijk = μ + Di + Sj + Ak + (D × S)ij + eijk(1)

where Yijk is the dependent variable, μ is the overall mean, Di is the effect of dietary treatment (i = 3; CON, AC, and ACMAN), Sj is the effect of sampling time (j = 3 for BW, j = 2 for B-HBA, antioxidant enzymes activities, total antioxidant capacity, oxidative stress biomarkers, relative gene expressions, and j = 6 for milk yield and milk chemical composition), Ak is the animal’s random effect, (D × S)ij is the interaction between the dietary treatments and sampling time, and eijk is the residual errors.

In the BW and B-HBA datasets, one-way ANOVA was also applied to determine the differences of the different sampling measurement times independently (start, lambing, and end for ewes BW and prepartum and postpartum for B-HBA, respectively).

Dataset homogeneity was evaluated through Lavene’s test. Furthermore, the Shapiro–Wilk test was used to test the normality of our dataset. Post hoc analysis was carried out considering the Tukey multiple range test for the data that did not violate the homogeneity and normality tests. The significance threshold was set at 0.05 while *p* ≥ 0.05 ≤ 0.10 was considered as a tendency. GraphPad Prism 8.4.2. was used for the interleaved bars and error bars represent the standard error mean (SEM).

## 3. Results

### 3.1. Lambing Features and BW

CON ewes of both breeds (27 in total) gave birth to 52 lambs (1.93 prolificacy), 27 of them being females and 25 males. More specifically, 23 lambs (13 females and 10 males; 1.92 prolificacy) were from Chios, while 29 lambs (14 females and 15 males; 1.93 prolificacy) were from the Lacaune breed. Those of the AC group (29 in total) gave birth to 54 lambs (1.86 prolificacy), 28 females and 24 males. More precisely, 32 lambs (18 females and 14 males; 2.00 prolificacy) were from Chios, while 22 lambs (11 females and 11 males; 1.69 prolificacy) were from the Lacaune breed. Ewes of the ACMAN group (26 ewes) gave birth to 48 lambs (1.85 prolificacy), 30 of them being females and 18 males. In detail, 26 lambs (17 females and 9 males; 1.86 prolificacy) were from Chios, while 22 lambs (13 females and 9 males; 1.83 prolificacy) were from the Lacaune breed.

For both breeds, the recorded BW did not significantly change (*p* > 0.05) between the dietary treatments (Table 3 and Table S2). Furthermore, the resulting BW re-gain (BWG) from the lambing to the final day of the experiment was found to be significantly higher (*p* = 0.013) in the ACMAN Chios ewes, while for the Lacaune ewes, was tended to increase in the ACMAN ewes compared to the AC ewes (*p* = 0.085) (Table 3). The effect of breed and its interactions are presented in Table S3.

### 3.2. Blood β-Hydroxybutyric Concentration

In the prepartum period, the ACMAN-fed ewes had significantly lower (Chios; *p* = 0.039 and Lacaune; *p* = 0.030) β-hydroxybutyric (B-HBA) acid concentrations in their blood compared to the CON (Table 4). Additionally, AC-Lacaune ewes also had significantly lower (*p* = 0.030) B-HBA in blood, compared to the CON (Table 4). Furthermore, in the postpartum period, B-HBA concentration was reduced (Chios; *p* = 0.001 and Lacaune; *p* = 0.068) in both the AC and ACMAN ewes compared to the CON (Table 4). Considering the repeated measures analysis, the overall results of the B-HBA concentration (both pre-and postpartum and both ewes’ breeds) were also significantly reduced (*p* < 0.001) in the AC and ACMAN (Table S4). The effect of breed and its interactions are presented in Table S5.

### 3.3. Milk Yield and Milk Chemical Composition

Chios ewes milk performance was not significantly affected (Table 5). On the other hand, milk yield (kg/d), and ECM (kg/d) were significantly increased (*p* = 0.001, *p* = 0.017, *p* = 0.006, respectively) in both AC and ACMAN Lacaune ewes (Table 5). The increased milk yield in AC and ACMAN Lacaune ewes resulted in higher fat, protein, casein, and lactose yield (*p* = 0.058, *p* = 0.001, *p* = 0.003, and *p* = 0.001, respectively) compared to the CON group (Table 5).

As for the sampling time, significant differences (*p* < 0.01) were observed in milk yield (kg/d), FCM (kg/d), and ECM (kg/d) for both Chios and Lacaune ewes, with the highest being recorded in the fourth sampling week for both breeds. Furthermore, a significant increase (*p* < 0.001) in fat (%) was observed for the Chios ewes in the first sampling week, while fat yield (g/d) was at the highest level (*p* < 0.001) in the second sampling week. Protein and casein yield (g/d) were significantly higher (*p* = 0.001 and *p* = 0.003, respectively) in the fourth and fifth sampling weeks, lactose (%) (*p* < 0.001) and non-fat solids (%) (*p* < 0.001) in the fifth week, lactose yield (g/d) (*p* = 0.001) in the fourth week, and total solids (*p* < 0.001) in the first week.

Regarding the Lacaune ewes, fat (%), fat yield (g/d), and protein (%) were significantly higher (*p* < 0.001, *p* < 0.001, and *p* = 0.011) in the fourth sampling week, while protein yield (g/d) (*p* < 0.001), and solids not fat (%) (*p* < 0.001) in the fifth week. Casein (%), casein yield (g/d), lactose (%), and lactose yield (g/d) were significantly higher (*p* = 0.014, *p* < 0.001, *p* = 0.004, and *p* < 0.001, respectively) in the fifth week, while total solids (%) were significantly higher (*p* < 0.001) in the first sampling week. The effect of breed and its interactions are presented in Table S6.

### 3.4. Blood Plasma and Milk Oxidative Status

The results for antioxidant enzyme activities, total antioxidant capacity, and oxidative stress biomarkers in blood plasma and milk are presented in Table 6. In blood plasma, the activity of superoxide dismutase (SOD) significantly increased (*p* = 0.037) in the ACMAN compared to the CON (Table 6). Furthermore, there was a trend for an increase in the activity of glutathione peroxidase (GSH-Px) of the AC group compared to the CON, while the activities of catalase (CAT), glutathione reductase (GR), and glutathione transferase were not significantly affected (Table 6). In addition, total antioxidant capacity measured by the [2,2′-Azino-bis (3-ethylbenzthiazoline-6-sulfonic acid)] ABTS assay was significantly increased (*p* = 0.038) in the ACMAN compared to the CON and significantly increased in the sixth week compared to the third (*p* < 0.001), also demonstrating a significant interaction between treatment and sampling time (*p* < 0.001) (Table 6). MDA significantly decreased (*p* = 0.006) in the sixth week compared to the third, while protein carbonyls significantly increased (*p* = 0.001) in the sixth week compared to the third (Table 6).

Regarding milk oxidative status, the total antioxidant capacity measured by the ABTS assay was significantly increased (*p* = 0.021) in the AC and ACMAN compared to the CON (Table 6). Moreover, protein carbonyls (PC) were significantly reduced (*p* = 0.023) in the AC and ACMAN compared to the CON (Table 6). The activities of SOD, CAT, and LPO did not significantly differ (Table 6). As for the sampling time, CAT, LPO, FRAP, and MDA were significantly reduced (*p* < 0.05) in the sixth compared to the third week.

### 3.5. Ewes Immune Transcriptional Profile

**Figure 1:**
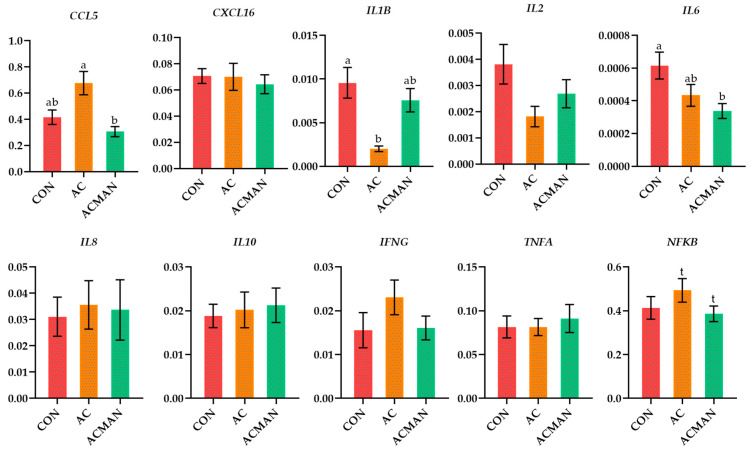
The relative transcript levels of selected genes in Chios ewes’ monocytes of the three dietary treatment groups (CON, AC, and ACMAN). Means with different superscript letters (a, b) differ significantly at *p* < 0.05. Error bars represent the SEM. CCL5, C-X-C motif chemokine 5; CXCL16, C-X-C motif chemokine ligand 16; INFG, interferon γ; IL1B, interleukin-1 beta; IL2, interleukin-2; IL6, interleukin-6; IL8, interleukin-8; IL10, interleukin-10; TNFA, tumor necrosis factor A; NFKB, nuclear factor kappa B.

**Figure 2:**
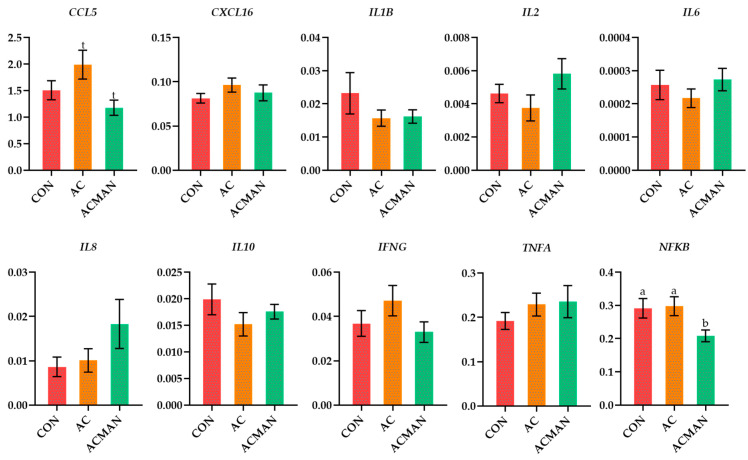
The relative transcript levels of selected genes in Chios ewes’ neutrophils of the three dietary treatment groups (CON, AC, and ACMAN). Means with different superscript letters (a, b) differ significantly at *p* < 0.05, and t is referred to values between 0.05 and 0.100 (0.05 < t < 0.100). Error bars represent the SEM. CCL5, C-X-C motif chemokine 5; CXCL16, C-X-C motif chemokine ligand 16; INFG, interferon γ; IL1B, interleukin-1 beta; IL2, interleukin-2; IL6, interleukin-6; IL8, iterleukin-8; IL10, interleukin-10; TNFA, tumor necrosis factor A; NFKB, nuclear factor kappa B.

The relative transcript levels of the selected genes related to innate immunity in monocytes and neutrophils are presented in Figure 1 and Figure 2 and Table S7. In monocytes, the relative transcript levels of the *CCL5* were significantly downregulated (*p* = 0.007) in the ACMAN compared to the AC while were significantly higher (*p* = 0.001) in the sixth compared to the third week (Figure 1 and Table S7). Furthermore, the relative transcript levels of the *IL1B* were significantly downregulated (*p* = 0.004) in monocytes of the AC compared to the CON, while the relative transcript levels of the *IL6* were significantly downregulated (*p* = 0.026) in the ACMAN compared to the CON (Figure 1 and Table S7). Finally, there was a trend for downregulation in the relative transcript levels of the *NFKB* in the ACMAN compared to the AC and were significantly higher (*p* < 0.001) in the sixth compared to the third week (Figure 1 and Table S7).

Regarding neutrophils, a trend was reported in the relative transcript levels of the *CCL5* in the ACMAN compared to the AC, while the relative transcript levels of the *NFKB* were significantly downregulated (*p* = 0.020) in the ACMAN compared to the CON and AC and were significantly higher (*p* = 0.007) in the sixth compared to the third week (Figure 2 and Table S7).

## 4. Discussion

Up to now, the effect of probiotic live yeasts (LY), postbiotics, and organic Se-enriched yeast supplementation on animal physiology, productivity, and health-related parameters has been sufficiently documented. However, this is the first study that investigates the effect of the combined supplementation of pro- and postbiotics in ewes’ production characteristics, milk quality, as well as energy, oxidative, and innate immunity status during the peripartum period in farm scale condition under the influence of commercial farm sanitary levels and common challenges.

### 4.1. Supplementation of LY and Postbiotics Blend Improved Ewes’ Energy Status during the Peripartum Period

As already stated, small ruminants may face a NEB during the peripartum period due to the high animal requirements, prolificacy, and stressful environmental and physiological conditions. Their demands for energy and nutrients are increased, while dry matter intake is decreased. To manage and adapt to the increased energy demands, NEFA from body fat reserves and glucose sparing for lactogenesis are mobilized [31]. This mobilized body fat due to NEB increases the concentration of NEFA and B-HBA in the blood [32]. Hence, B-HBA has been already used as a marker of excessive NEB in dairy ewes during the peripartum period [13] and during the transition period of dairy cows [31]. The reported threshold of B-HBA in sheep with subclinical ketosis varies from 0.5 mmol/1 to 1.6 mmol/1 [33,34,35], and that for those with clinical ketosis varies from 1.6 mmol/1 to 7 mmol/1. Previous results in healthy ewes of the same flock that were supplemented with 2 g LY/ewe/day agree with the findings of this study [13]. However, Dunière et al. [5] reported that as parturition approached, although B-HBA concentrations were found to be reduced in ewes of the yeast-supplemented group, this reduction was not statistically significant, while NEFA concentrations were increased in both the control and the yeast-supplemented group. The lower values of B-HBA in the blood of treated ewes of our study indicate a more efficient nutrient utilization in the rumen, which may improve overall metabolic status in both breeds and especially in Lacaune, for which production (milk yield) was improved.

### 4.2. Supplementation of LY and Postbiotics Blend Improved Ewes’ Performance

Ewes from the three groups fully recovered their BW from the lambing period to the end of the experiment. Interestingly, in the ACMAN group in both breeds, the significantly increased BWG may be attributed to the combination of LY, MOS, and organic selenium. In compliance with our results, lambs fed with a combination of LY plus MOS improved average daily gain, feed efficiency, and dietary energy compared to the LY-supplemented and the control lambs [14]. Grossi et al. [36] also reported improved final weight and daily average gain when supplementing beef cattle with LY, MOS, and organic Se. Furthermore, Tassinari et al. [37] also reported a higher BWG when calves were administered MOS during the first phase of the cattle-fattening cycle. In addition, administering selenium-enriched yeast led to improved growth performance in beef cattle [38].

It is noteworthy that the supplemented yeast products significantly affected milk performance for the Lacaune but not for the Chios ewes, which should also be considered for an accurate evaluation of the results. This may be rendered to the fact that Lacaune ewes have higher yield than Chios, which may trigger physiological mechanisms at different rates, resulting in significant discrepancies in the different breeds. In a recent meta-analysis study in which *Saccharomyces cerevisiae* was assessed in small ruminants’ milk yield and milk chemical composition, the breed was one of the limiting factors that explained most of the milk yield variability [39]. Our results concerning the impact of LY on Chios ewes’ milk performance reported high consistency since the milk yield was only numerically increased in our previous work supplementing the same commercial product [13]. Considering the difference between the breeds, it could be hypothesized that these supplements may be more efficient in high-genetic-merit animals, for which the importance of nutrient management, rumen function, and GIT homeostasis are more critical. Improving the gastrointestinal environment by supplementing LY and MOS beneficially affects rumen integrity and consequently animal performance. The former further signifies the different responses of animals with diverse genetics and performance under certain feeding strategies when they are fed as an integrated flock. It can be assumed that MOS and/or beta-glucans eventually reach the gastrointestinal tract, improving mammary health and milk production [40]. The beneficial effect of supplementing yeast products on small ruminants’ productivity has already been justified in previous studies [13,41,42]. More specifically, the inclusion of 14 g of yeast culture (*Saccharomyces cerevisiae*)/ewe/day increased the milk net energy content, total solids, and milk fat and protein content. Stella et al. [41] reported an increase (14%) in goat milk yield as well. Regarding dairy cows, it has been proven that milk yield was beneficially affected when LY was administered in early [43], mid [44], and late lactation [45]. Moreover, Dawson and Tricarico [46] gathered the outcomes of 22 different studies and concluded that milk yield was increased by 7.3% when diets were supplemented with LY.

### 4.3. Supplementation of LY and Postbiotics Blend Triggered Antioxidant Mechanisms in Chios Ewes

Increased energy and nutrient requirements may expose imbalances, causing lipid mobilization and/or blood hyperketonemia, thus inducing animals’ oxidative stress [47]. In the present study, Chios ewes were selected not only to validate our previously published results [13] but also to more deeply investigate the effects of different LY products and the combination of yeasts’ mannans, beta-glucans, and organic Se supplementation on ewes’ oxidative status postpartum. In this study, antioxidant mechanisms were more effectively responded to the ACMAN group (increased SOD activity and total antioxidant capacity measured by the ABTS method), on which B-HBA concentration in Chios ewes was found at the lowest levels pre- and postpartum. The increased activity of SOD in the ACMAN group is of paramount importance since SOD is the first line of the organism’s defense against ROS [48]. SOD catalyzes the dismutation of superoxide anion free radical (O_2-_) into molecular oxygen and H_2_O_2_ and reduces the O_2_^−^ level that is linked with cell damage at excessive concentrations [49]. In a previous study of our group, supplementing 2 g LY/ewe/day significantly increased the total antioxidant capacity measured through the ABTS method [13].

Another parameter that might benefit the antioxidant response in the ACMAN dietary treatment could also be the Se source supplementation (inorganic vs. organic) and consequently its bioavailability. It has been well documented that organic Se has greater bioavailability from rumen microflora, and thus for the host, than its inorganic form [50]. Gong et al. [22] found increased GSH-Px activity and total antioxidant capacity in dairy cows that were offered 0.3 mg selenised yeast/kg for 60 days as compared to cows fed sodium selenite at the same level. Novoselec et al. [51] reported increased GSH-Px and SOD activity and lower MDA levels in the blood of late-gestation Se-fed ewes compared to the unsupplemented ones [52]. Likewise, lower MDA values, higher GSH-Px activity, and higher total antioxidant capacity were reported in the blood of pregnant goats that were supplemented with Se. In addition, in the muscles of the Se-supplemented growing goats, total antioxidant capacity and GSH-Px activity were increased, while MDA was found at lower levels [53]. Similarly, Alimohamady et al. [54] reported considerably increased GSH-Px activity in the blood of Se-supplemented (organic or inorganic) lambs compared to the unsupplemented ones, and Mousaie [55] reported low levels of MDA and high GSH-Px activity in growing lambs supplemented with 1.2 mg Se (organic or inorganic) compared to the unsupplemented ones.

Milk oxidative stability was beneficially affected in both the AC and ACMAN groups as reflected by a lower concentration of protein carbonyls and higher antioxidant potential as measured with ABTS. In our previous work, total antioxidant capacity measured with the ABTS method also significantly increased when LY was supplemented in ewes’ during the peripartum period [13]. Moreover, milk Se bioavailability has also been proven to be potent. Significantly higher Se concentration was found in the milk [51,56] and colostrum [56] of Se-supplemented ewes. Improving oxidative stability may unveil the potential of yeast-based products to improve milk quality and self-life longevity.

### 4.4. Supplementation of LY and Postbiotics Blend Unveiled Important Findings Regarding Chios Ewes’ Innate Immunity

Supplementing LY products and MOS has been considered an efficient nutritional strategy for maintaining gut health which not only supports better animal productivity but also preserves animal health [19,57,58,59]. It should not be overlooked that the immune system is strongly related to a healthy gastrointestinal tract [60,61], and the use of pro- and prebiotics is a powerful ally in this direction [62]. Regarding the relative transcript levels of the studied genes related to innate immunity, our results suggest that the differences in the relative transcript levels of the studied genes could slightly benefit the ACMAN ewes compared to those of the CON and AC. More specifically, the two most prevalent proinflammatory cytokines, TNF-α and interleukin-1 (IL-1β), are induced by nuclear factor kappa b (NF-kB) and mitogen-activated protein kinase (MAPK). In our study, *IL1B* relative transcript levels were downregulated in the AC group, which is an important finding since it controls B-cell maturation and proliferation, activates natural killer cells, and is linked to the acute manifestation of inflammation in immune cells [63]. Furthermore, *NFKB,* which was downregulated in the neutrophils of the ACMAN, contributes to the regulation of inflammasomes and stimulates the expression of several proinflammatory genes, including those that produce cytokines and chemokines [64]. Moreover, *NFKB* is essential for controlling innate immune cells’ and inflammatory T cells’ survival, activation, and differentiation [64]. Another studied gene that was downregulated in this study was *IL6* in monocytes, which is also an important proinflammatory cytokine [65]. Therefore, the outcomes of this study may indicate a positive impact of yeast products and postbiotics on ewes’ innate immunity during the peripartum period. However, these results require further evaluation and validation under challenging conditions, also investigating the expression of corresponding proteins. Proinflammatory chemokines and cytokine downregulations may be related to the decreased levels of the B-HBA that were reported in the AC and ACMAN groups [17]. In our previous work, supplementing 2 g LY/ewe/day significantly reduced the relative transcript levels of the *CCL5*, *CXCL16*, and *IL8* in ewes’ monocytes and reduced the relative transcript levels of the IL10 in ewes’ neutrophils during the peripartum period.

Due to their anti-mutagenic and antioxidant properties, MOS can prevent harmful bacteria from adhering to the intestinal mucosa, strengthening it and supporting the immune system [17]. Furthermore, animals’ innate immune systems can be strengthened by beta-glucans, probably partly because immune cells such as macrophages have receptors for 1,3- and 1,6-branched glucans [18]. However, the quantity of beta-glucans and MOS bypassing the rumen must be better understood [40]. Therefore, it has been proven that MOS promote the growth of beneficial bacteria, limit the propagation of intestinal pathogens, reduce the prevalence of gastrointestinal disorders, and can promote animal immunity [57,62,66,67,68]. Another benefit of MOS supplementation worth mentioning is the fact that it has even been proven to boost calves’ immunity due to the supplementation in cows’ diets [57]. Furthermore, Diaz et al. [19] suggested that in high-concentration diets fed to beef cattle supplemented with either LY or MOS, ruminal pH is increased, LPS translocation into the bloodstream is decreased, and blood SAA concentration is decreased. These factors support that these products could be supplemented to improve the rumen environment in beef cattle, which is susceptible to ruminal subacute acidosis, and reduce the inflammation caused by the consumption of grain-based diets.

In addition, the immunoregulatory role of Se has already been well documented [21]. The administered Se source may play a role in inflammatory response through the modulation of cytokine production. The results in the ACMAN group may manifest the potential of organic Se supplementation compared to inorganic supplementation in situations of physiological stress. Inorganic Se sources may reveal limited bioavailability due to antagonistic interactions within the gastrointestinal tract [69]. The use of organic Se has been introduced as an effective strategy due to reduced interactions, particularly within the rumen, and lower formation of unavailable elemental Se [50]. Therefore, organic Se may be more bioavailable than inorganic Se to ruminants and supports a greater extent of oxidative status and innate immunity than the use of inorganic Se sources.

## 5. Conclusions

The improvement of animal performance and energy efficiency via the consumption of live yeast alone or in combination with postbiotics was more prominent and effective in the high-milk-yielding ewes. The combination of live yeast with postbiotics and organic Se advanced immune oxidative status of Chios ewes.

## Figures and Tables

**Table 1:** Experimental dietary schedule of the three dietary treatments (CON, AC, and ACMAN) before and after parturition as fed.

	Dietary Treatments ^1^		
Feed Intake (ewe/day)	CON	AC	ACMAN
Before parturition			
Alfalfa hay, kg	0.7	0.7	0.7
Oat hay, kg	0.7	0.7	0.7
Concentrate ^a^, kg	1.00	1.00	1.00
Live yeast ^b^, g	-	1.00	1.00
Mannans and beta-glucans ^c^, g	-	-	2.00
Inorganic Selenium (Se) ^d^, mg	0.30	0.30	-
Organic Selenium (Se) ^e^, mg	-	-	0.20
After parturition			
Alfalfa hay, kg	0.7	0.7	0.7
Oat hay, kg	0.7	0.7	0.7
Concentrate ^a^, kg	2.00	2.00	2.00
Live yeast ^b^, g	-	1.00	1.00
Mannans and beta-glucans ^c^, g	-	-	2.00
Inorganic Selenium (Se) ^d^, mg	0.60	0.60	-
Organic Selenium (Se) ^e^, mg	-	-	0.40

^1^ CON, control dietary treatment; AC, dietary treatment supplemented with ActiSaf^®^ and inorganic selenium; ACMAN, dietary treatment supplemented with ActiSaf^®^, Safmannan^®^, and SelSaf^®^.^a^ 57.5% maize grain, 18% wheat middlings, 22% soybean meal, 2.5% mineral and vitamins premix. ^b^ ActiSaf^®^, ^c^ Safmannan^®^, ^d^ sodium selenite, ^e^ SelSaf^®^.

**Table 2:** Experimental feeds (concentrates and forages) chemical composition and nutrient intake in each dietary group before and after parturition.

	Concentrates ^1^			Forages	
Composition, %	CON	AC	ACMAN	Oat hay	Alfalfa hay
Dry matter, as-fed basis	88.63	88.60	88.87	87.60	88.16
Ash, DM basis	4.98	5.33	5.34	7.64	8.31
Crude protein, DM basis	18.90	19.36	18.79	8.40	16.74
Ether extract, DM basis	2.53	2.37	2.40	2.28	1.59
aNDFom ^2^, DM basis	17.73	17.36	17.33	61.23	49.09
ADF, DM basis	7.24	7.09	6.97	44.21	36.98
	CON	AC		ACMAN	
Prepartum nutrients intake, g/ewe/day					
Dry matter	2117.0	2116.7		2119.4	
Ash	142.2	145.3		145.6	
Crude protein	322.3	326.3		321.8	
Ether extract	46.5	45.1		45.4	
aNDFom ^2^	835.7	832.7		832.6	
ADF	563.4	562.0		561.1	
Postpartum nutrients intake, g/ewe/day					
Dry matter	3002.9	3002.3		3007.7	
Ash	186.3	192.5		193.1	
Crude protein	489.8	497.8		488.8	
Ether extract	68.6	65.8		66.4	
aNDFom ^2^	992.6	986.0		986.4	
ADF	627.7	624.9		623.1	

^1^ CON, control dietary treatment; AC, dietary treatment supplemented with ActiSaf^®^ and inorganic selenium; ACMAN, dietary treatment supplemented with ActiSaf^®^, Safmannan^®^, and SelSaf^®^. ^2^ aNDFom, ash-free neutral detergent fiber treated with amylase. Nutrient intake was registered on a group basis, and for that, SEM values are not presented.

**Table 3:** Analysis of variance in Chios and Lacaune ewes’ body weight (BW) at the start of the experiment, at lambing, and at the end and the body weight gain from lambing to the end.

	CON	AC	ACMAN	SEM	*p*-Value
Chios ewes					
BW, kg, prepartum	67.8	67.3	73.0	3.30	0.423
BW, kg, lambing	65.2	64.4	69.6	3.16	0.470
BW, kg, end	68.4	67.8	74.3	3.10	0.283
BW gain, kg (lambing–end)	3.3 ^b^	3.6 ^b^	4.7 ^a^	0.57	0.013
Lacaune ewes					
BW, kg, prepartum	76.5	74.7	84.4	3.45	0.152
BW, kg, lambing	72.7	72.1	80.6	3.34	0.171
BW, kg, end	76.7	75.3	85.3	3.37	0.112
BW gain, kg (lambing–end)	4.1	3.2	4.6	0.40	0.085

CON, control dietary treatment (12 Chios and 15 Lacaune); AC, dietary treatment supplemented with ActiSaf^®^ and inorganic selenium (16 Chios and 13 Lacaune); ACMAN, dietary treatment supplemented with ActiSaf^®^, Safmannan^®^, and SelSaf^®^ (14 Chios and 12 Lacaune). Means with different superscripts (^a^, ^b^) between dietary treatments differ significantly (*p* < 0.05). SEM: Standard error of the mean. A significance level below 0.05 indicates a significant difference.

**Table 4:** Analysis of variance in β-hydroxybutyric (B-HBA) acid at prepartum and postpartum of the Chios and Lacaune ewes of the three dietary treatments.

	CON	AC	ACMAN	SEM	*p*-Value
Chios ewes					
B-HBA, mmol/L, Prepartum	0.89 ^a^	0.65 ^ab^	0.58 ^b^	0.08	0.039
B-HBA, mmol/L, Postpartum	0.65 ^a^	0.46 ^b^	0.48 ^b^	0.04	0.001
Lacaune ewes					
B-HBA, mmol/L, Prepartum	0.91 ^a^	0.55 ^b^	0.57 ^b^	0.08	0.030
B-HBA, mmol/L, Postpartum	0.61	0.42	0.43	0.05	0.068

CON, control dietary treatment (12 Chios and 15 Lacaune); AC, dietary treatment supplemented with ActiSaf^®^ and inorganic selenium (16 Chios and 13 Lacaune); ACMAN, dietary treatment supplemented with ActiSaf^®^, Safmannan^®^, and SelSaf^®^ (14 Chios and 12 Lacaune). Means with different superscripts (^a^, ^b^) between dietary treatments differ significantly (*p* < 0.05). SEM: standard error of the mean. A significance level below 0.05 indicates a significant difference.

**Table 5:** Milk yield, FCM6%, ECM, and milk chemical composition from Chios and Lacaune ewes of the three dietary treatments (CON, AC, and ACMAN) throughout the experimental period (1st, 2nd, 3rd, 4th, 5th, and 6th experimental weeks).

	Dietary Treatment (D) ^1^				Sampling Time (S) in Weeks							Effect (D × S)		
	CON	AC	ACMAN	SEM	1st	2nd	3rd	4th	5th	6th	SEM	D	S	D × S
Chios ewes														
Milk Yield, kg/d	2.25	2.28	2.43	0.06	2.18 ^C^	2.40 ^AB^	2.18 ^C^	2.47 ^A^	2.40 ^AB^	2.28 ^AB^	0.16	0.885	0.004	0.818
FCM_6%_, kg/d	2.50	2.37	2.59	0.26	2.57 ^AB^	2.84 ^A^	2.17 ^C^	2.82 ^A^	2.07 ^C^	2.46 ^B^	0.16	0.822	<0.001	0.572
ECM, kg/d	2.10	2.02	2.20	0.23	2.12 ^B^	2.33 ^A^	1.88 ^C^	2.34 ^A^	1.89 ^C^	2.08 ^B^	0.14	0.851	<0.001	0.623
Fat, %	7.09	6.70	6.75	0.34	7.70 ^A^	7.58 ^A^	6.40 ^B^	7.31 ^A^	5.18 ^C^	6.89 ^AB^	0.28	0.697	<0.001	0.401
Fat yield, g/d	145.0	144.4	159.5	15.43	163.4 ^D^	180.3 ^DE^	130.5 ^B^	157.4 ^CD^	114.4 ^A^	151.9 ^CD^	10.17	0.734	<0.001	0.006
Protein, %	5.07	4.99	5.04	0.09	5.00	4.99	4.98	5.00	5.11	5.12	0.08	0.858	0.473	0.684
Protein yield, g/d	115.0	113.6	122.2	13.87	110.7 ^B^	120.9 ^AB^	108.6 ^B^	123.6 ^A^	122.6 ^A^	115.4 ^AB^	8.28	0.893	0.001	0.634
Casein, %	3.94	3.88	3.93	0.09	3.90	3.86	3.90	3.91	3.96	3.98	0.07	0.849	0.500	0.471
Casein yield, g/d	88.3	88.7	95.1	10.43	86.0 ^B^	92.0 ^AB^	85.0 ^B^	96.0 ^A^	95.0 ^A^	90.2 ^AB^	6.25	0.875	0.003	0.644
Lactose, %	4.92	5.01	4.98	0.06	4.80 ^D^	4.91 ^CD^	5.02 ^B^	4.98 ^BC^	5.10 ^A^	5.01 ^B^	0.05	0.573	<0.001	0.524
Lactose yield, g/d	111.2	115.8	121.0	13.97	105.5 ^D^	117.6 ^BC^	110.4 ^CD^	123.8 ^A^	123.5 ^AB^	115.2 ^BCD^	8.42	0.891	0.001	0.934
Total solids, %	17.65	16.59	16.84	0.45	18.30 ^A^	17.70 ^AB^	16.29 ^C^	17.39 ^B^	15.51 ^D^	17.00 ^B^	0.33	0.250	<0.001	0.021
Solids not fat, %	10.57	10.61	10.62	0.08	10.57 ^AB^	10.49 ^B^	10.54 ^B^	10.52 ^B^	10.79 ^A^	10.71 ^A^	0.05	0.893	<0.001	0.256
Lacaune ewes														
Milk Yield, kg/d	2.14 ^b^	3.51 ^a^	3.32 ^a^	0.27	2.68	2.91 ^C^	2.98 ^BC^	3.17 ^A^	3.13 ^A^	3.08 ^AB^	0.19	0.001	<0.001	0.377
FCM_6%_, kg/d	2.29 ^b^	3.32 ^a^	3.14 ^ab^	0.27	2.84 ^C^	3.11 ^B^	2.68 ^CD^	3.42 ^A^	2.60 ^D^	2.85 ^C^	0.18	0.017	<0.001	0.003
ECM, kg/d	1.92 ^b^	2.91 ^a^	2.74 ^a^	0.22	2.38 ^BC^	2.61 ^B^	2.37 ^BC^	2.86 ^A^	2.39 ^BC^	2.52 ^B^	0.16	0.006	<0.001	0.030
Fat, %	6.50	5.85	5.68	0.29	6.65 ^A^	6.67 ^A^	5.40 ^B^	6.90 ^A^	4.81 ^C^	5.64 ^B^	0.26	0.112	<0.001	0.003
Fat yield, g/d	141.2 ^t^	194.7 ^t^	184.4	16.73	174.0 ^C^	191.5 ^B^	154.2 ^CD^	211.4 ^A^	143.5 ^D^	165.8 ^C^	11.86	0.058	<0.001	0.003
Protein, %	4.88	4.98	4.77	0.11	4.84 ^BC^	4.82 ^C^	4.81 ^C^	4.80 ^C^	5.00 ^A^	4.98 ^AB^	0.08	0.404	0.011	0.878
Protein yield, g/d	103.0 ^b^	174.5 ^a^	160.5 ^c^	13.50	128.4 ^C^	140.7 ^B^	143.2 ^B^	152.8 ^A^	156.7 ^A^	154.1 ^A^	9.58	0.001	<0.001	0.338
Casein, %	3.75	3.85	3.72	0.11	3.73 ^BC^	3.75 ^BC^	3.71 ^C^	3.70 ^C^	3.88 ^A^	3.86 ^AB^	0.07	0.713	0.014	0.940
Casein yield, g/d	81.2 ^b^	134.9 ^a^	125.7 ^a^	11.33	100.1 ^D^	110.6 ^C^	111.4 ^C^	118.7 ^AB^	122.5 ^A^	120.5 ^A^	7.78	0.003	<0.001	0.307
Lactose, %	4.94 ^b^	5.21 ^a^	5.11 ^a^	0.05	5.01 ^C^	5.06 ^C^	5.09 ^BC^	5.07 ^C^	5.18 ^A^	5.12 ^AB^	0.04	0.001	0.004	0.356
Lactose yield, g/d	106.3 ^b^	182.8 ^a^	170.4 ^a^	13.93	135.4 ^D^	148.0 ^C^	152.6 ^BC^	161.3 ^A^	162.9 ^A^	158.9 ^AB^	10.00	0.001	<0.001	0.297
Total solids, %	16.90	17.01	16.99	0.35	17.91 ^A^	17.20 ^BC^	16.22 ^D^	17.66 ^AB^	15.83 ^D^	16.98 ^C^	0.28	0.967	<0.001	0.444
Solids not fat, %	10.48	10.77	10.62	0.10	10.66 ^BC^	10.44 ^D^	10.54 ^CD^	10.54 ^CD^	10.80 ^A^	10.76 ^AB^	0.07	0.108	<0.001	0.388

^1^ CON, control dietary treatment (12 Chios and 15 Lacaune); AC, dietary treatment supplemented with ActiSaf^®^ and inorganic selenium (16 Chios and 13 Lacaune); ACMAN, dietary treatment supplemented with ActiSaf^®^, Safmannan^®^, and SelSaf^®^ (14 Chios and 12 Lacaune). Means with different superscripts (a, b) between dietary treatments and (A, B, C, D, E) between sampling times differ significantly (*p* < 0.05), and t indicates a trend toward a significant difference (0.05 < t < 0.100). FCM_6%_ = fat-corrected milk in 6% calculated by the equation FCM_6%_ = (0.28 + 0.12 × F) × MY, where F = milk fat % and MY = milk yield in kg. ECM = energy-corrected milk yield calculated by the equation ECM = milk yield × (0.071 × milk fat (%) + 0.043 × milk protein (%) + 0.2224). SEM: standard error of the mean. Effect: the dietary treatment (D), sampling time (S), and the interaction between dietary treatment × time (D × S). A significance level below 0.05 indicates a significant difference.

**Table 6:** Enzyme activities (Units/mL), total antioxidant capacity, and oxidative status biomarkers in blood plasma and milk of Chios ewes of the three dietary treatments (CON, AC, ACMAN) at two sampling weeks (3rd and 6th).

	Dietary Treatments (D) ^1^				Sampling Time (S)			Effect ^2^		
	CON	AC	ACMAN	SEM	3rd	6th	SEM	D	S	D × S
Blood Plasma										
Superoxide dismutase, Units/mL	47.83 ^b^	49.10 ^ab^	52.05 ^a^	1.102	48.57	50.72	1.010	0.037	0.127	0.090
Catalase, Units/mL	28.50	27.93	28.49	0.451	28.21	28.40	0.340	0.609	0.630	0.014
Glutathione peroxidase, Units/mL	0.13 ^t^	0.17 ^t^	0.15	0.012	0.16	0.15	0.010	0.082	0.247	0.004
Glutathione reductase, Units/mL	0.02	0.02	0.02	0.002	0.02	0.02	0.001	0.546	0.233	0.670
Glutathione transferase, Units/mL	0.062 ^t^	0.064	0.077 ^t^	0.005	0.09	0.04	0.004	0.068	<0.001	0.175
ABTS ^3^, % inhibition	24.74 ^b^	25.64 ^ab^	28.14 ^a^	0.882	28.47 ^A^	23.88 ^B^	0.770	0.034	<0.001	0.002
FRAP ^4^, μM ascorbic acid	0.48	0.46	0.57	0.040	0.48	0.52	0.040	0.115	0.544	0.453
Malondialdehyde, μM	1.00	0.99	1.09	0.053	1.10 ^A^	0.95 ^B^	0.040	0.321	0.006	0.051
Protein carbonyls, nmol/mL	3.35 ^t^	3.07 ^t^	3.32	0.100	2.99 ^B^	3.50 ^A^	0.090	0.129	0.001	0.886
Milk										
Superoxide dismutase, Units/mL	63.38	63.83	66.80	2.12	66.53	62.81	1.65	0.475	0.114	0.397
Catalase, Units/mL	4.53	4.04	4.22	0.42	4.55 ^A^	3.97 ^B^	0.25	0.702	0.022	0.134
Lactoperoxidase, Units/mL	0.55	0.68	0.43	0.10	0.64 ^A^	0.46 ^B^	0.08	0.216	0.037	0.141
ABTS ^3^, % inhibition	24.11 ^b^	27.88 ^a^	27.81 ^a^	1.00	27.36	25.84	0.94	0.021	0.282	0.562
FRAP ^4^, μM ascorbic acid	2.27	2.34	2.47	0.10	2.54 ^A^	2.18 ^B^	0.11	0.377	0.008	0.001
Malondialdehyde, μM	0.54 ^t^	0.48	0.46 ^t^	0.03	0.56 ^A^	0.43 ^B^	0.03	0.163	0.001	0.060
Protein carbonyls, nmol/mL	2.75 ^b^	2.24 ^a^	2.10 ^a^	0.16	2.18 ^B^	2.54 ^A^	0.12	0.023	0.015	0.646

^1^ CON, control dietary treatment (8 Chios ewes); AC, dietary treatment supplemented with ActiSaf^®^ and inorganic selenium (8 Chios ewes); ACMAN, dietary treatment supplemented with ActiSaf^®^, Safmannan^®^, and SelSaf^®^ (8 Chios ewes). ^2^ Effect, the dietary treatments (D), sampling time (S), and the interactions between dietary treatments × time (D × S). ^3^ ABTS: 2,20-Azino-bis (3-ethylbenzthiazoline-6-sulfonic acid as % inhibition. ^4^ FRAP: ferric-reducing ability of plasma is expressed as μM ascorbic acid equivalents. Means with different superscript letters (^a^, ^b^) between dietary groups and (A, B) between sampling time points differ significantly at *p* < 0.05, and t is referred to values between 0.05 and 0.100 (*p* ≥ 0.05 ≤ 0.10).

## Data Availability

The datasets used and/or analyzed during the current study are available from the corresponding author upon reasonable request.

## Supplementary Materials

The following supporting information can be downloaded at: https://www.mdpi.com/article/10.3390/jof9121139/s1, Table S1. Sequences, amplicon size, and annealing temperature of the primers designed to be specific for *Ovis Aries* and were used in real-time qPCR. Table S2. Body weight (BW) of the Chios and Lacaune ewes of the three dietary treatments (CON, AC, ACMAN) throughout the experimental period (Before parturition, after partition, and final day of the experiment). Table S3. Body weight (BW) of the ewes of the three dietary treatments (CON, AC, and ACMAN) throughout the experimental period (start of the experiment (P), at lambing (L), and the end (E) of the experiment). Table S4. The β-hydroxybutyric acid (B-HBA) in Chios and Lacaune ewes’ blood prepartum and postpartum from ewes of the three dietary treatments (CON, AC, ACMAN) in 1–3 weeks prepartum, and 1–3 weeks postpartum. Table S5. Blood β-hydroxybutyric acid (B-HBA) in ewes of the three dietary treatments (CON, AC, and ACMAN) in 1–3 weeks prepartum and 1–3 weeks postpartum. Table S6. Milk yield, fat-corrected milk yield (FCM6%), energy-corrected milk yield (ECM), and milk chemical composition from ewes of the three dietary treatments (CON, AC, and ACMAN) throughout the experimental period (1st, 2nd, 3rd, 4th, 5th, and 6th experimental week). Table S7. The relative transcript levels of genes in blood monocytes and neutrophils of ewes fed the three diets (CON, AC, ACMAN) at two sampling times (3rd and 6th week postpartum).
