# Supplementary material for: The Effect of Dietary Supplementation with Probiotic and Postbiotic Yeast Products on Ewes Milk Performance and Immune Oxidative Status
Source: J Fungi (Basel). 2023 Nov 25;9(12):1139. doi: 10.3390/jof9121139 (PMC10744422; doi:10.3390/jof9121139)
Supplement: Supplementary file 1 [file jof-09-01139-s001.zip › jof-2687111-supplementary.pdf]

The Effect of Dietary Supplementation with Blend of Probiotic & Postbiotic Yeast Products on Dairy Ewes Milk Performance and Immune-Oxidative Status during the Peripartum Period

Christos Christodoulou[1], Alexis Skourtis[1], Panagiota Kyriakaki[1], Fotis Fokion Satolias[1], Dimitris Karabinas[1], Maxime Briche[2], Nizar Salah[2], George Zervas[1], Alexandros Mavrommatis[1], Eleni Tsiplakou[1],*

[1] Laboratory of Nutritional Physiology and Feeding, Department of Animal Science, School of Animal Biosciences, Agricultural University of Athens, Iera Odos 75, GR-11855 Athens, Greece
[2] Phileo by Lesaffre, Marcq en Baroeul, 59700 Nord, France
* Correspondence: eltsiplakou@aua.gr

Table S1. Sequences, amplicon size, and annealing temperature of the primers designed to be specific for *Ovis Aries* and were used in real-time qPCR.

Gene	Acc. No.	bp	Tm °C	Forward primer 5′-3′	Reverse primer 5′-3′
CCL5	XM_027975305	61	61	CAAGTGCTCCATGGCAGCAG	GTTGGCGCACACCTGACG
CXCL16	XM_015098600	70	60	GTGCCTGTGTTGTCCCTCTT	GCTTGCACACCACGTAGAGT
IL1B	NM_001009465	70	60	TGGATAGCCCATGTGTGCTG	CAGAACACCACTTCTCGGCT
IL2	NM_001009806	70	60	AAATCCCGAGAACCTCAAGCT	TGTAGCGTTAACCTTGGGCA
IL6	NM_001009392	61	60	CAGCAAGGAGACACTGGCAGA	TCCATCTTTTTCCTCCATTTTTGG
IL8	NM_001009401	71	60	CCTGCTCTCTGCAGCTCTGTG	TGCATTGGCATCGAAGTTCTG
IL10	NM_001009327	100	60	CTGGGGGAGAAGCTGAAGAC	CTCTCTTCACCTGCTCCACC
IFNG	NM_001009803	146	60	AAATTCCGGTGGATGATCTG	ACCATTACATTGATGCTCTCC
TNFA	NM_001024860	70	60	GGGAGACACAAACTAAGGGCT	AACCTGCAGTTCAGCTCCG
NFKB	XM_042251202	70	60	AAGCTGTGGTGGAGGACTTG	ACAGAGTTACCCAAGCGGTC
GAPDH	NM_001190390	75	62	AAAGGCCATCACCATCTTCCA	ACCACGTACTCAGCACCAGCAT
YWHAZ	NM_001267887	70	62	TGTTCTATTGTGCCTAGTACACTGT	CATCAAGACTCACTGCCTCCC

Table S2. Body weight (BW) of the Chios and Lacaune ewes of the three dietary treatments (CON, AC, ACMAN) throughout the experimental period (Before parturition, after partition, and final day of the experiment).

	Dietary Treatments (D)				Time (T)				Effect		
Chios											
	CON	AC	ACMAN	SEM	Pre.	Post.	End	SEM	D	S	D×S
BW, kg	67.1	66.5	72.2	1.1	69.4^{B}	66.4^{C}	70.2^{A}	1.4	0.39	<0.001	0.03
Lacaune											
	CON	AC	ACMAN	SEM	Pre.	Post.	End	SEM	D	S	D×S
BW, kg	67.1	66.5	72.2	3.4	78.5^{B}	75.1^{C}	79.1^{A}	2.0	0.14	<0.001	0.17

Means with different superscripts (A, B, C) between sampling times differ significantly ($P < 0.05$).
SEM: Standard error of the mean.
Effect: The dietary treatment (D), measurement time (T), and the interaction between dietary treatment x time (D x T).
A significance level below 0.05 indicates a significant difference.

Table S3. Body weight (BW) of the ewes of the three dietary treatments (CON, AC, and ACMAN) throughout the experimental period (start of the experiment (P), at lambing (L), and the end (E) of the experiment).

	Dietary Treatments (D)				Sampling Time (S)				Effect[b]					
	CON	AC	ACMAN	SEM[a]	P	L	E	SEM[a]	D	S	D×S	B	B×S	B×S×D
BW (kg)	71.7	69.9[t]	77.4[t]	0.900	73.8[B]	70.6[C]	74.5[A]	1.400	0.090	<0.001	0.010	0.002	0.438	0.507

Means with different superscripts (A, B, C) between sampling times differ significantly at P < 0.05 and t is referred to values between 0.05 and 0.100 (0.05< t < 0.100)

[a]SEM: Standard error of the mean.

[b]Effect: The dietary treatment (D), the breed (B), the sampling time (S), and the interaction between dietary treatment x time (D x S) breed and sampling time (B x S) and dietary treatment, breed, and sampling time (D x B x S). CON: control dietary treatment; AC: dietary treatment supplemented with ActiSaf® and inorganic selenium; ACMAN: dietary treatment supplemented with ActiSaf®, Safmannan®, and SelSaf®.

Table S4. The β-hydroxybutyric acid (B-HBA) in Chios and Lacaune ewes' blood prepartum and postpartum from ewes of the three dietary treatments (CON, AC, ACMAN) in 1 - 3 weeks prepartum, and 1 - 3 weeks postpartum.

	Dietary Treatment (D)				Sampling Time (S)			Effect		
					Chios					
	CON	AC	ACMAN	SEM	1st	2nd	SEM	D	S	D×S
B-HBA, mmol/L	0.77[a]	0.56[b]	0.53[b]	0.04	0.71[B]	0.53[A]	0.04	0.005	<0.001	0.447
					Lacaune					

	CON	AC	ACMAN	SEM	1st	2nd	SEM	D	S	D×S
B-HBA, mmol/L	0.76[a]	0.49[b]	0.50[b]	0.07	0.68[A]	0.49[B]	0.05	0.007	0.006	0.477

Means with different superscript (a, b) between dietary treatments and (A, B) between sampling times differ significantly (P < 0.05).
SEM: Standard error of the mean.
Effect: The dietary treatment (D), sampling time (S), and the interaction between dietary treatment x time (D x S).
A significance level below 0.05 indicates a significant difference.

Table S5. Blood β-hydroxybutyric acid (B-HBA) in ewes of the three dietary treatments (CON, AC, and ACMAN) in 1 - 3 weeks prepartum and 1 - 3 weeks postpartum.

	Dietary Treatment (D)				Sampling Time (S)			Effect[b]						
	CON	AC	ACMAN	SEM	1st	2nd	SEM	D	S	D×S	B	B×S	B×S×D
B-HBA (mmol/L)	0.76[a]	0.53[b]	0.51[b]	0.020	0.69[A]	0.51[B]	0.030	<0.001	<0.001	0.245	0.430	0.278	0.804

Means with different superscripts (a, b) between dietary treatments and (A, B) between sampling times differ significantly at P < 0.05.
[a]SEM: Standard error of the mean.
[b]Effect: The dietary treatment (D), the breed (B), the sampling time (S), and the interaction between dietary treatment x time (D x S) breed and sampling time (B x S) and dietary treatment, breed, and sampling time (D x B x S). CON: control dietary treatment; AC: dietary treatment supplemented with ActiSaf® and inorganic selenium; ACMAN: dietary treatment supplemented with ActiSaf®, Safmannan®, and SelSaf®

Table S6. Milk yield, fat corrected milk yield ($FCM_{6\%}$), energy corrected milk yield (ECM), and milk chemical composition from ewes of the three dietary treatments (CON, AC, and ACMAN) throughout the experimental period (1st, 2nd, 3^{d}, 4th, 5th, and 6th experimental week).

	Dietary Treatment (D)				Sampling time (S)							Effect (D×S)						
	CON	AC	ACMAN	SEM	1st	2nd	3^{d}	4th	5th	6th	SEM	D	S	D×S	B	B×S	B×S×D
Milk Yield, kg/d	2.19^{b}	2.83^{a}	2.84^{a}	0.050	2.40^{C}	2.62^{B}	2.53^{BC}	2.79^{A}	2.74^{C}	2.65^{B}	0.130	0.041	<0.001	0.592	0.003	0.046	0.646
$FCM_{6\%}$, kg/d	2.39	2.80	2.85	0.050	2.67^{C}	2.94^{B}	2.41^{D}	3.09^{A}	2.32^{A}	2.63^{B}	0.120	0.191	<0.001	0.156	0.050	0.233	0.031
ECM, kg/d	2.00	2.42	2.45	0.040	2.22^{BC}	2.44^{B}	2.10^{C}	2.58^{A}	2.12^{C}	2.28^{B}	0.100	0.116	<0.001	0.288	0.027	0.091	0.127
Fat, %	6.76	6.32	6.25	0.080	7.17^{A}	7.14^{A}	5.95^{B}	7.12^{A}	5.01^{C}	6.28^{B}	0.200	0.197	<0.001	0.279	0.008	0.413	0.043
Fat yield, g/d	147.8	167.0	171.0	8.310	166.7^{B}	184.0^{A}	141.5^{C}	192.8^{A}	129.1^{D}	157.4^{B}	7.930	0.341	<0.001	0.089	0.137	0.353	0.012
Protein, %	4.96	4.99	4.91	0.020	4.92^{AB}	4.91^{B}	4.90^{B}	4.90^{B}	5.05^{A}	5.05^{A}	0.060	0.743	0.019	0.689	0.093	0.515	0.519
Protein yield, g/d	108.3^{t}	140.9^{t}	139.9	2.650	117.8^{D}	129.0^{BC}	123.9^{CD}	136.6^{A}	137.9^{A}	133.1^{AB}	6.460	0.057	<0.001	0.209	0.012	0.008	0.872
Caseins, %	3.83	3.86	3.84	0.020	3.81^{AB}	3.80^{B}	3.80^{B}	3.80^{B}	3.92^{A}	3.92^{A}	0.050	0.949	0.021	0.658	0.081	0.579	0.707
Caseins yield, g/d	84.4^{t}	109.4^{t}	109.3^{t}	2.080	91.7^{C}	100.1^{B}	96.6^{BC}	106.2^{A}	107.5^{A}	104.0^{AB}	5.080	0.050	<0.001	0.221	0.011	0.021	0.785
Lactose, %	4.93^{a}	5.10^{b}	5.04^{a}	0.010	4.90^{D}	4.98^{CD}	5.05^{BC}	5.02^{BC}	5.13^{A}	5.06^{BC}	0.030	0.014	<0.001	0.279	0.011	0.285	0.734
Lactose yield, g/d	108.5	145.9	143.8	2.760	118.6^{C}	131.1^{B}	129.2^{B}	140.7^{A}	141.4^{A}	135.2^{AB}	6.740	0.026	<0.001	0.500	0.002	0.206	0.795
Total solids, %	17.23	16.78	16.91	0.100	18.04^{A}	17.44^{BC}	16.25^{D}	17.50^{B}	15.67^{E}	16.95^{C}	0.220	0.517	<0.001	0.151	0.858	0.243	0.067
Solids not fat, %	10.52	10.69	10.62	0.020	10.61^{B}	10.47^{C}	10.53^{BC}	10.53^{BC}	10.78^{A}	10.73^{A}	0.050	0.181	<0.001	0.043	0.780	0.807	0.909

Means with different superscript (a, b) between dietary treatments and (A, B, C, D) between sampling times differ significantly at $P < 0.05$, and t is referred to values between 0.05 and 0.100 ($0.05 < t < 0.100$).

$FCM_{6\%}$ = Fat corrected milk in 6 % calculated by the equation $FCM_{6\%} = (0.28 + 0.12 \times F) \times MY$, where F = milk fat % and MY = milk yield in kg.

ECM = Energy corrected milk yield calculated by the equation ECM = milk yield × (0.071 × Milk Fat (%) + 0.043 × Milk Protein (%) + 0.2224)

[a]SEM: Standard error of the mean.

[b]Effect: The dietary treatment (D), the breed (B), the sampling time (S), and the interaction between dietary treatment x time (D x S) breed and sampling time (B x S) and dietary treatment, breed, and sampling time (D x B x S). CON: control dietary treatment; AC: dietary treatment supplemented with ActiSaf® and inorganic selenium; ACMAN: dietary treatment supplemented with ActiSaf®, Safmannan®, and SelSaf®.

Table S7. The relative transcript levels of genes in blood monocytes and neutrophils of ewes fed the three diets (CON, AC, ACMAN) at two sampling times (3rd and 6th week postpartum).

	Dietary treatments (D)[1]				Sampling time (S)			Effect[2]		
Genes[3]	CON	AC	ACMAN	SEM	3rd	6th	SEM	Diet	Time	D×S
					Monocytes					
CCL5	0.42[ab]	0.68[a]	0.31[b]	0.04	0.37[B]	0.56[A]	0.06	0.007	0.001	0.358
CXCL16	0.07	0.07	0.06	0.005	0.07	0.06	0.006	0.819	0.357	0.603
IL1B	0.010[a]	0.002[b]	0.008[ab]	0.001	0.007	0.006	0.001	0.004	0.767	0.941
IL2	0.004	0.002	0.003	0.0004	0.002	0.003	0.0007	0.216	0.096	0.851
IL6	0.0006[a]	0.0004[ab]	0.0003[b]	0.00004	0.0004	0.0005	0.00006	0.026	0.142	0.702
IL8	0.03	0.04	0.03	0.005	0.04	0.03	0.008	0.96	0.797	0.089
IL10	0.02	0.02	0.02	0.002	0.02	0.02	0.003	0.915	0.718	0.081
IFNG	0.02	0.02	0.02	0.002	0.02	0.02	0.003	0.409	0.111	0.021
TNFA	0.08	0.08	0.08	0.007	0.09	0.08	0.01	0.873	0.213	0.463
NFKB	0.41	0.49[t]	0.39[t]	0.03	0.30[B]	0.56[A]	0.03	0.089	<0.001	0.073
					Neutrophils					
CCL5	1.51	1.99[t]	1.18[t]	1.13	1.45	1.67	1.77	0.079	0.273	0.676
CXCL16	0.08	0.10	0.09	0.004	0.08[B]	0.10[A]	0.006	0.478	0.003	0.502
IL1B	0.023	0.016	0.016	0.002	0.016	0.021	0.003	0.334	0.336	0.545
IL2	0.005	0.004	0.006	0.0005	0.004[B]	0.005[A]	0.0007	0.297	0.036	0.499
IL6	0.0003	0.0002	0.0003	0.00002	0.0003[A]	0.0002[B]	0.00003	0.672	0.009	0.432
IL8	0.01	0.01	0.02	0.002	0.02[t]	0.01[t]	0.003	0.229	0.051	0.476
IL10	0.02	0.015	0.018	0.001	0.018	0.017	0.002	0.505	0.405	0.865
IFNG	0.04	0.05	0.03	0.003	0.05[A]	0.03[B]	0.003	0.283	<0.001	0.478
TNFA	0.19	0.23	0.24	0.02	0.17[B]	0.27[A]	0.03	0.430	0.001	0.062
NFKB	0.29[a]	0.30[a]	0.21[b]	0.02	0.23[B]	0.31[A]	0.02	0.020	0.007	0.201

[1]CON, control dietary treatment; AC, dietary treatment supplemented with ActiSaf® and inorganic selenium; ACMAN, dietary treatment supplemented with ActiSaf®, Safmannan®, and SelSaf®.

[2]Effect, the dietary treatments (D), the breed (B), the sampling time (S), and the interactions between the dietary treatments × time (D×S).

[2]*CCL5*, *C-X-C motif chemokine 5*; *CXCL16*, *C-X-C motif chemokine ligand 16*; *INFG*, *Interferon γ*; *IL1B*, *Interleukin-1 beta*; *IL2*, *Interleukin-2*; *IL6*, *Interleukin-6*; *IL8*, *Interleukin-8*; *IL10*, *Interleukin-10*; *TNFA*, *Tumor Necrosis Factor A*; *NFKB*, *Nuclear Factor kappa B*.

Means with different superscript letters (a, b) differ significantly at $P < 0.05$, and t is referred to values between 0.05 and 0.100 (0.05 < t < 0.100).
